# Tuning the Morphological Properties of Granular Hydrogels to Control Lymphatic Capillary Formation

**DOI:** 10.1002/admi.202401037

**Published:** 2025-04-24

**Authors:** Daniel Montes, Sanjoy Saha, Angela Taglione, Donghyun Paul Jeong, Liao Chen, Fei Fan, Hsueh-Chia Chang, Donny Hanjaya-Putra

**Affiliations:** Bioengineering Graduate Program, University of Notre Dame, Notre Dame, IN 46556, USA; Chemical and Biomolecular Engineering, University of Notre Dame, Notre Dame, IN 46556, USA; Bioengineering Graduate Program, University of Notre Dame, Notre Dame, IN 46556, USA; Aerospace and Mechanical Engineering, University of Notre Dame, Notre Dame, IN 46556, USA; Chemical and Biomolecular Engineering, University of Notre Dame, Notre Dame, IN 46556, USA; Bioengineering Graduate Program, University of Notre Dame, Notre Dame, IN 46556, USA; Aerospace and Mechanical Engineering, University of Notre Dame, Notre Dame, IN 46556, USA; Chemical and Biomolecular Engineering, University of Notre Dame, Notre Dame, IN 46556, USA; Bioengineering Graduate Program, University of Notre Dame, Notre Dame, IN 46556, USA; Aerospace and Mechanical Engineering, University of Notre Dame, Notre Dame, IN 46556, USA; Bioengineering Graduate Program, University of Notre Dame, Notre Dame, IN 46556, USA; Chemical and Biomolecular Engineering, University of Notre Dame, Notre Dame, IN 46556, USA; Aerospace and Mechanical Engineering, University of Notre Dame, Notre Dame, IN 46556, USA; Bioengineering Graduate Program, University of Notre Dame, Notre Dame, IN 46556, USA; Chemical and Biomolecular Engineering, University of Notre Dame, Notre Dame, IN 46556, USA; Aerospace and Mechanical Engineering, University of Notre Dame, Notre Dame, IN 46556, USA; Center for Stem Cell and Regenerative Medicine, University of Notre Dame, Notre Dame, IN 46556, USA

**Keywords:** biomaterials, granular hydrogels, hyaluronic acid, lymphangiogenesis, lymphatic endothelial cells, porous biomaterials

## Abstract

Granular hydrogels show great promise in biomedical applications by mimicking the extracellular matrix and fostering a supportive microenvironment for tissue regeneration. This study investigates how tuning granular hydrogel properties influences lymphatic tube formation. Microgels were fabricated using norbornene-modified hyaluronic acid (NorHA) via pipetting or vortexing for 90 s (V90s) and 180 s (V180s), then assembled into granular hydrogels under loose and tight packing conditions. These conditions produced gels with varied pore morphologies and bulk rheological properties. Lymphatic capillary formation occurred only in tightly packed gels, where mechanical properties converged, highlighting the importance of gel morphology over stiffness. V180s samples showed earlier vessel formation as seen in lymphatic gene and protein expression, while pipetted gels exhibited greater capillary connectivity, forming larger vessel clusters and fewer small satellite structures. The pipetting gels also supported lower-curvature, more linear capillary networks that bridged multiple droplets, likely due to reduced entrapment in large voids compared to vortexed gels. These findings suggest that in bulk granular gels, lymphatic tube formation is governed not by mechanical stiffness but by pore size and gel topology (periodicity). Understanding and optimizing these morphological parameters can inform future strategies in lymphatic tissue engineering and regenerative medicine.

## 1. Introduction

Granular hydrogels, formed from microgel subunits, enhance cell proliferation by mimicking the extracellular matrix (ECM) and creating a physiologically relevant microenvironment, surpassing traditional 2D cell cultures.^[1–6]^ These sub-fractioned gels enable tissue constructs at various scales, supporting organoid production,^[7,8]^ and serving as building blocks for larger constructs.^[6,9,10]^ Hence, these materials have been used to produce scaffolds and bioinks in 3D bioprinting processes.^[2,3,7,8,11]^ Due to their granular structure, these materials offer large interstitial spaces, promoting the free movement of microgel subfractions.^[1,4]^ This feature improves injectability due to reduced stiffness compared to bulk hydrogels, while facilitating excellent microstructure recovery through self-healing.^[3]^ Moreover, their inherent porous structure enhances endogenous cell invasion, which is crucial for wound healing and tissue repair.^[12–15]^

Moreover, granular hydrogels can be engineered to support various 3D microenvironments by incorporating motifs that enhance cell proliferation, degradability, and therapeutic effects, facilitating improved tissue remodeling.^[6,12,16–18]^ Functionalization of the polymer backbones with RGD has shown enhanced cell adhesion and tissue morphogenesis,^[19–22]^ while the integration of degradable peptide sequences, such as matrix metalloproteinase (MMP)-sensitive sequences, modifies the scaffold stiffness and promotes remodeling.^[6,22–24]^

The porosity of granular hydrogels can be controlled by altering microgel size distribution^[25]^ and microgel packing (jamming).^[26,27]^ There are physical methods, such as centrifugation or vacuum packing, that can modulate the pore interconnectivity. Pore sizes can be further modified through intermolecular and chemical interactions. In this sense, Anderson et al.,^[28]^ Griffin et al.,^[12]^ and Liu et al.,^[14]^ have produced microparticles interconnection via covalent bonding to produce microporous scaffolds. Furthermore, Riley et al.,^[29]^ demonstrated that by using the same interparticle connectivity strategy, the micropore sizes are influenced by the size distribution of the microgels. Moreover, Widener et al.^[30–32]^ have used guest–host supramolecular assemblies to produce dynamic interparticle interactions to control the gel’s porosity while preserving the gel’s injectability. Additional studies have explored the implementation of interstitial matrices to create composite granular hydrogels for enhancing cell support and attachment in the micropores.^[3,6,10,22,33]^

The variable porosity of granular gels allows for the fabrication of different tissue constructs. For instance, porous architecture supports angiogenesis and vasculogenesis.^[34]^ Qazi et al.^[6]^ demonstrated that embedded human umbilical vein endothelial cells (HUVECs) spheroids can sprout within composite granular hydrogels, and that the length and density of sprouts is driven by the pore size. Similarly, Muir et al.,^[33]^ showed that cells undergo sprouting when the interstitial matrix is functionalized with RGD. Also, Ramirez-Calderon et al.^[10]^ used composite granular hydrogels made with peptide sequences to evaluate angiogenesis in vitro. The authors showed that the cells adapted the shape of the individual microgels and coated them prior to the formation of vessels. However, successful vascularization typically requires matrix support either through interstitial matrix or cell encapsulation within individual microgels.^[35,36]^ Moreover, typically cells from mesodermal lineage are co-cultured with the endothelial cells to provide additional support to the vasculature.^[6,10,22,37]^ However, there is limited understanding of how growth factors and matrix composition influence lymphatic vascular growth, which can explain the relatively few successful results in lymphatic endothelial cells (LEC) encapsulation in 3D scaffolds.^[38–41]^ There is also a lack of studies that explore the implementation of granular gels for supporting lymphangiogenesis.^[42–45]^

Lymphatic vessels perform a diverse set of physiological functions such as lymph circulation, inflammation modulation, and wound healing, while also playing a major role in the immune response to several pathologies.^[42,43,45–48]^ Hence, engineering lymphatic vessels is of special interest to produce tissue constructs with physiological fidelity.^[39,49–51]^ In this context, Rütsche et al.^[44]^ explored how granulated hydrogels systems can sustain lymphatic tube formation. The authors showed that granular hydrogels with different porosities had different outcomes on lymphangiogenesis with lower porosity leading to better tube formation outcomes due to the increased surface contact between microgels and the mechanical support provided to the lymphatic endothelial cells (LECs). However, it is still unclear how the different porosities affect lymphangiogenesis.^[52]^

Since LECs uniquely express lymphatic vessel endothelial hyaluronan receptor 1 (LYVE-1),^[53,54]^ we have previously shown that hyaluronic acid (HA) can not only preserve key lymphatic markers, but also promote lymphatic tube formation.^[55–57]^ By optimizing the parameters of norbornene-modified HA (NorHA) hydrogels, we can generate functional and mature lymphatic networks.^[58]^ Building upon previous studies, this work aims to employ composite granular hydrogels where the granular component serves as a template to provide mechanical stability to support lymphangiogenesis. Here, we evaluate the influence of the granular component morphology on lymphatic vasculature formation. The study also presents one of the first successful approaches to 3D in vitro production of lymphatic vessels without mesodermal lineage co-culture. We utilize NorHA to fabricate composite granular hydrogels, which include a non-degradable granular phase and a degradable interstitial matrix. Various granular gel morphologies were produced using different fabrication methods and packing degrees, and their rheological behavior and capacity to support lymphatic sprouting were evaluated.

## 2. Results and Discussion

### 2.1. NorHA Synthesis/Characterization and Granular Hydrogels Fabrication

**Figure 1.**
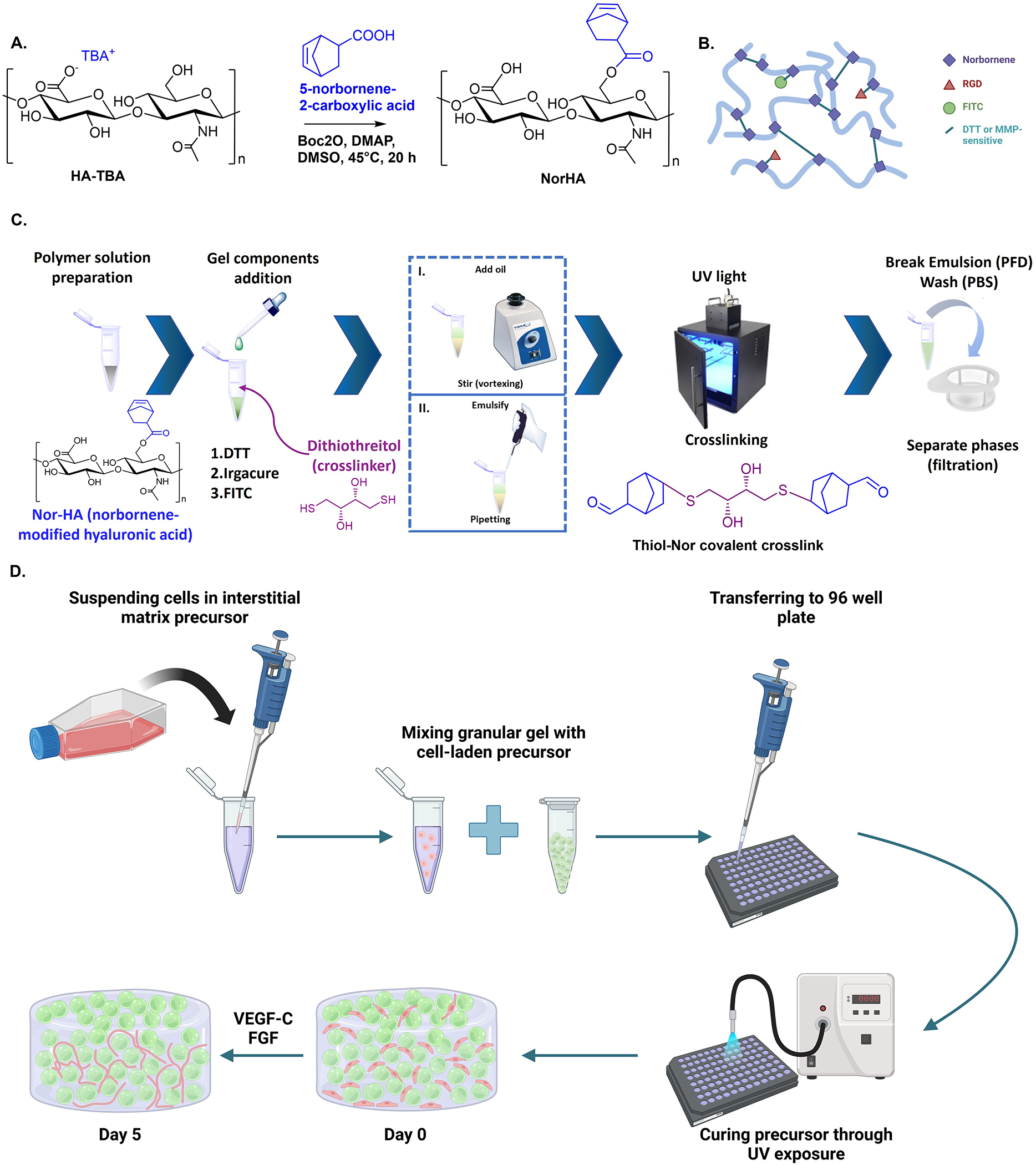
Graphical representation of granular hydrogels composites fabrication and lymphatic sprouting assays. A) Reaction mechanism from tetrabutylammonium hyaluronic acid (TBA-HA) to norbornene-modified hyaluronic acid (NorHA). B) NorHA crosslinking and functionalization with RGD and FITC using DTT or MMP-sensitive crosslinkers. C) Procedure followed for granular gels fabrication via vortexing and pipetting. D) Inclusion of a degradable interstitial matrix made with NorHA for supporting LECs sprouting.

As reported in previous studies, NorHA (Figure 1A) was used to fabricate granular gels.^[4,6,25,33,58]^ NorHA was specifically chosen for this study to take advantage of its HA backbones for LECs binding via their LYVE-1 receptor,^[53]^ interaction which has been shown in the past to upregulate key lymphatic markers and preserve lymphatic phenotypes.^[55,57,59,60]^ The degree of substitution (DS) of norbornene groups on the HA backbone was estimated via ^1^H-NMR, obtaining 22% DS (Figure S1, Supporting Information). Norbornene allows for an easy crosslinking process via thiol-ene reactions with dithiothreitol (DTT) and MMP-sensitive crosslinkers modified with thiol groups (Figure 1B). At the same time, the norbornene-modified HA backbone can be easily functionalized with other motifs for gel visualization purposes (i.e., FITC), and for enhancing cells attachment to the ECM (i.e., RGD). In this sense, the polymer solution formulation was adjusted to use 80% of norbornene groups for crosslinking and 1% by FITC.

Different methods were utilized for fabricating the granular gels (Figure 1C), namely vortexing and a new pipetting method recently developed in our lab.^[61,62]^ By using a pipette with an elliptical cross-section to enhance the destabilizing azimuthal curvature and capillary pressure, the pinching action becomes more robust and periodic resulting in uniformly sized droplets. We expected to obtain diverse granular gels morphologies by using these different methods,^[25]^ which would give insights on their effect on different properties of the hydrogels such as their rheological behavior, cell invasion, and lymphatic tube formation. We also evaluated the abilities of the different granular hydrogels to support lymphatic sprouting. Human LECs were suspended in an interstitial matrix precursor produced with NorHA, which would provide microgels interconnectivity while supporting the lymphatic sprouts (Figure 1D). 3D lymphatic tube formation was finally evaluated after five days of culture. Further results reported in this study are mainly divided into five sections: I) Morphological characterization of the granular gels, II) the effect of the gels’ morphology on their rheological behavior, III) lymphatic sprouting assays performed in granular hydrogels composites, IV) the evaluation of the lymphatic capillaries connectivity, and V) the influence of the gel morphology on the lymphatic gene expression.

### 2.2. Morphological Characterization of the Granular Hydrogels

Granular hydrogels were produced using different alternatives to test their effects on the morphological characteristics. For the pipetting alternative, an automated system incorporating a stepper motor for controlling solution dispensing was used (Figure S2, Supporting Information). Dispensing flow rate and dispensing/aspiration cycles were varied to determine their effect on the microgels’ uniformity. Results showed that for the evaluated range, the flow rate had no significant effect on the droplet size distribution, whereas higher number of cycles resulted in higher uniformity (Figure S3, Supporting Information). Hence, a flow rate of 0.66 μL s^−1^ and 20 dispensing/aspiration cycles were chosen for fabricating the granular gel at scale. Similarly, the effect of the vortexing duration on the size distribution of the microgels was studied (Figure S4, Supporting Information). Interestingly, by increasing the vortexing time, we observed that their size distribution became more uniform with lower average. Based on this, two different vortexing times (90 s and 180 s) were used for producing gel at scale, as the wider size distributions of gels may lead to tight microgels packing without changing the microgels jamming conditions.

**Figure 2.**
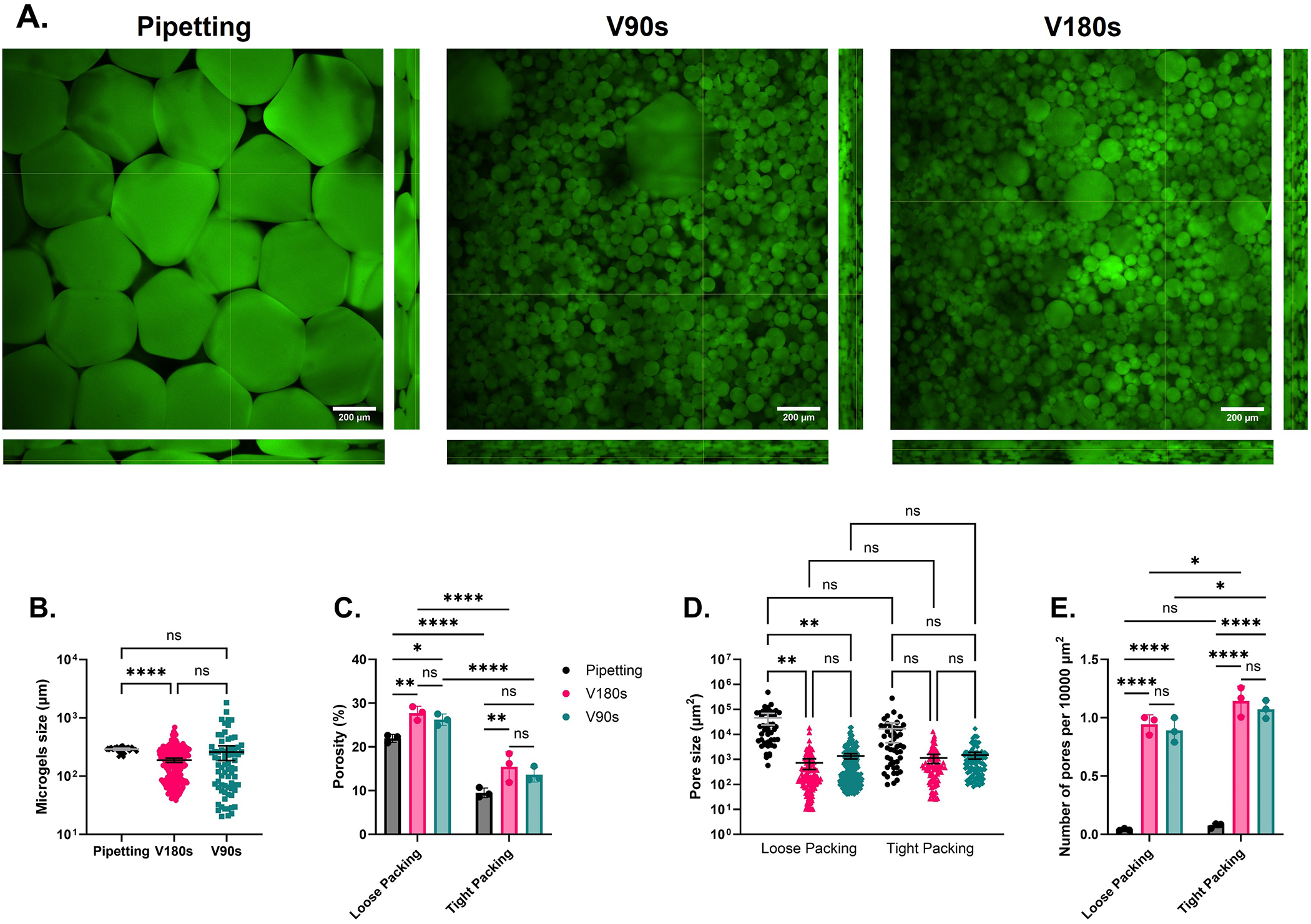
Morphological characterization of the granular gels produced by vortexing and pipetting. A) Orthogonal view of confocal imaging of FITC labeled granular gels, and porous structure rendering, respectively for Pipetting, Vortexing 90s (V90s), and Vortexing 180s (V180s). B) Scatter plot for the microgels sizes for each one of the granular hydrogels samples. C) Calculated porosity through ImageJ. D) Pore cross sectional area. E) Number of pores per 10 000 μm^2^ cross sectional area. Scale bar is 200 μm. The error bars represent the mean and CI (95%) for samples *n* >30 (B,C), while for *n* < 30 these represent the mean and standard deviation (C,D). *p < 0.05, **p < 0.01, ***p < 0.001, ****p < 0.0001.

Granular hydrogels were produced by microgels jamming via centrifugation at 1500 *g* (loose packing) and 6000 *g* (tight packing). Visually, the gels produced by vortexing and pipetting have different sizes for the microgels subunits and different porous morphologies (Figure 2A, and Figure S5A, Supporting Information). This may affect the interconnectivity within the granular gels and the kind of tissue structures that can be obtained.^[6]^ Moreover, it was also observed that each one of the samples has different size distributions (Figure 2C), with the granular gel prepared by vortexing for 90 s having the widest distribution, while pipetting method resulted in the most uniform distribution as expected.^[61,62]^

Additional analyses of the porous structure of the granular hydrogels were conducted. It was observed that there are significant differences in the porosity depending on the degree of packing of the microgels after the jamming process (Figure 2C). Additionally, the porosity of the vortexing samples was statistically similar, with significant differences with the pipetting-produced granular gel for both the loose and tight packing scenarios. The last had the lowest porosity (≈22% loose packing and ≈9% for tight packing); although, due to the different size dispersion of the microdroplets, each sample had a different pore size distribution for the loose packing condition (Figure 2D) which also resulted in larger pore diameter (Figure S5C, Supporting Information). Conversely, for the tightly packed gels those differences were not significant. This suggests that the packing process (i.e., centrifugation) have greater influence on the porous structure of the granular gels than the fabrication method and size distribution of the microgels.^[29]^ Moreover, the pores’ density was estimated (Figure 2E). As expected, lower pore count per sectional area (10000 μm^2^) were obtained for the pipetting sample due to its larger particles size.^[6]^ However, marginal differences were observed between the loose and tight packing scenarios, suggesting that higher centrifugation velocity affects the porosity but not the pores density.

These different interstitial properties can affect the mechanical properties of the granular gels, as with larger pores it is expected to have less interparticle friction.^[63]^ Hence, the rheological properties of the gels produced by both methods should be significantly different.^[6]^ Moreover, previously it has been described that the interstitial structure of the granular gels can act as a physical cue,^[28]^ affecting the phenotype of embedded cells and tissue development.^[14,15]^ Therefore, we expected variations in the LEC sprouting morphologies based on the granular gel type.

### 2.3. Effect of Morphology on the Rheological Behavior of the Hydrogels

**Figure 3.**
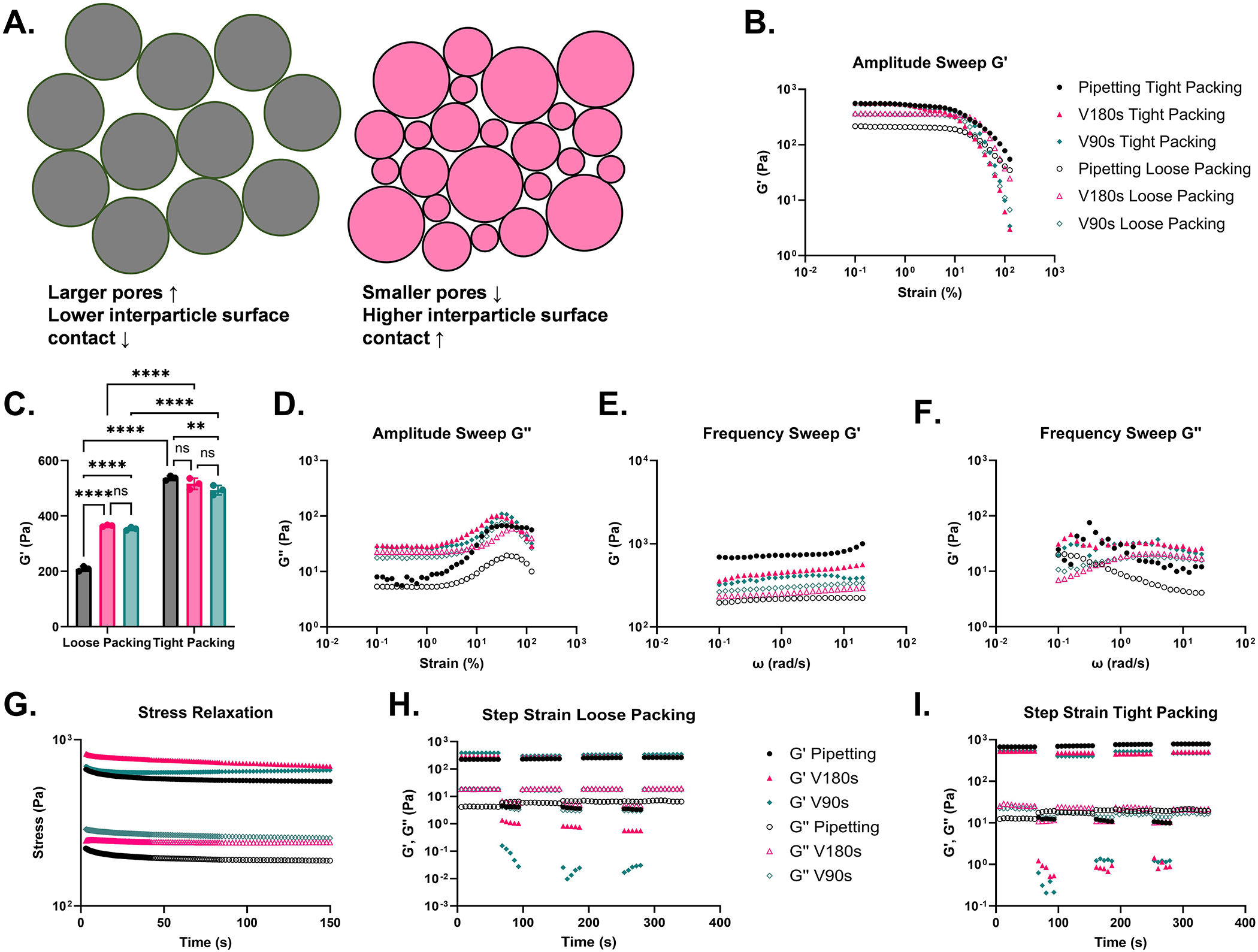
Rheological behavior evaluated for the granular gels fabricated at loose packing and tight packing conditions. A) Representation of interparticle surface contact changes with respect to the pore size. B) Amplitude sweep—*G*’ measured from 1% to 130% strain at 10 rad s^−1^. C) Elastic modulus estimated from the linear viscoelastic region (LVR) of the materials measured at 10 rad s^−1^. D) Amplitude sweep—*G*” measured from 1% to 130% strain at 10 rad s^−1^. E) Frequency sweep—*G*’ measured from 0.1 to 20 rad s^−1^ at 1% strain. F) Frequency sweep—*G*” measured from 0.1 to 20 rad s^−1^ at 1% strain. G) Stress relaxation estimated for the samples at 1% strain. H) Step strain measurements carried out using time ramps for 1% and 800% strain for loose packed gels, and time ramps for 1% and 400% for the tight packed gels. B–G) Filled symbols correspond to the tight packing condition while the hollow symbols correspond to the loose packing condition. H,I) Filled symbols correspond to the storage modulus (*G*’) while the hollow symbols correspond to the loss modulus (*G*”). All samples were measured by triplicate at 25 °C. The error bars represent the mean and standard deviation. *p < 0.05, **p < 0.01, ***p < 0.001, ****p < 0.0001.

It is expected that granular hydrogels with different morphological characteristics have different rheological behaviors as their elastic and viscous components are governed by their interparticle surface contact (i.e., friction) and deformation.^[63]^ Hence, for granular hydrogels composed of microgels with smooth surfaces, not only the porosity, but also the size of the pores plays an important role on their rheological behavior as these morphological properties determine the microgels’ contact (Figure 3A).^[27,63]^ Therefore, the effect of the granular hydrogels packing on their oscillatory rheological behavior was evaluated.

By evaluating the materials via amplitude sweep, we observed that the linear viscoelastic region (LVR) for the loose packing conditions was shorter for the pipetting-produced gel than that of both vortexing-produced gels (Figure S6A, Supporting Information). These results are in agreement with the pore sizes calculated for the materials, as with a higher packing the interparticle surface contact for the vortexing hydrogels is greater than for the pipetting gel due to their smaller pore sizes. Nonetheless, while at low strains the vortexing gels had a notably higher elastic behavior, their elastic modulus decayed faster than for the pipetting gel which is demonstrated by their crossover points indicating the shift towards a viscous-like behavior at a strain of ≈43% for the V90s and ≈91% for V180s. Interestingly, the crossover point was not observed for the pipetting gel within the experimental range, meaning that its predominant elastic behavior is preserved over a larger strain range. This last effect is presumably due to the dominance of the microgels’ mechanical response over the bulk material’s response due to their size distribution which is much larger than the hydrogel pores.^[63]^ Nonetheless, at tight packing conditions, the crossover point is clearly observed for all the samples, with the pipetting-produced gel remaining higher (Figure S6B, Supporting Information).

Similarly, the storage modulus for the tightly packed gels is significantly higher than the one of their loose packing counterparts (Figure 3B). Additionally, the storage modulus was similar across all the granular hydrogels fabricated under tight packing conditions. These findings are presumably related to the reduction in the porosity of the materials when using a higher centrifugation velocity, as the interparticle surface contact is increased, thereby increasing the contribution from the bulk material. We anticipate that these differences in the rheological behavior with the materials’ packing may have implications on lymphatic morphogenesis, as at loose packing conditions the mechanical support provided by the granular hydrogels would be lower. Moreover, as at static conditions the mechanical properties of the tight packed granular hydrogels are similar. Hence, we anticipate that the differences in the lymphatic sprouting can be attributed to the distinct materials morphology.^[6]^

The additional rheological characterization shows that the *G*’ of the frequency sweep tests for the loose packing condition (Figure 3D) suffers little to no variations with increasing the rate of deformation, while for the tight packing there is a small build-up at 12 rad s^−1^. For all the samples the loss modulus (*G*”) slightly reduces with the rate of deformation (Figure 3F). Furthermore, the samples show typical stress relaxation behavior with the initial modulus value following similar trends as from the previous tests (Figure 3G). The self-healing behavior of the gels was also evaluated. For all the loose packing samples (Figure 3H) there is a full mechanical recovery after being subjected to 800% strain. Conversely, at strains higher than 400% no reliable data for the storage modulus of the tightly packed sample was collected due to the sensitivity of the instrument. However, the materials experienced similar mechanical recovery after decreasing the strain (Figure 3I). As shown by the amplitude sweep tests, the difference between the elastic and viscous modulus at 800% (loose packing) and 400% (tight packing) strain for the pipetting-produced gel is smaller compared to the shift observed for the vortexing-produced gels, which again is in agreement with a predominant microgels deformation contribution over the pore structure re-shaping contribution at high strains.^[63]^

### 2.4. Lymphatic Tube Formation within Composite Granular Hydrogels

As reported before, the morphology of granular gels can affect the type of tissue-like architecture of constructs obtained when embedding cells in these interstitial spaces.^[6,44]^ Porosity can be especially important during early-stage of lymphatic vessel formation as the connectivity between tube fragments may be affected by microgels’ surface contact.^[6,64]^ Nonetheless, mechanical stability is needed to obtain lymphatic sprouting. This mechanical stability can be provided by supporting cells (co-culture) and/or by the inclusion of a synthetic ECM as has been reported in previous studies.^[44]^ In our preliminary studies we evaluated the sprouting capacity of lymphatic endothelial cells spheroids embedded within granular gels with no backfilling matrix (Figure S7, Supporting Information). However, no relevant sprouting was observed after culturing the cells for several days.

Hence, lymphatic tube formation was tested on the granular hydrogels by seeding human LECs on a degradable interstitial matrix precursor as shown in Figure 1D. The interstitial matrix formulation included 5 mm of RGD and 1.2 mm of MMP-sensitive crosslinkers.^[56,57]^ Such formulation was chosen based on a previous study in which the bulk NorHA composition was optimized for supporting functional and mature lymphatic networks.^[58]^ In addition, 100 ng mL^−1^ of VEGF-C and 50 ng mL^−1^ of FGF were used in the culture media to stimulate lymphatic tube formation.

**Figure 4.**
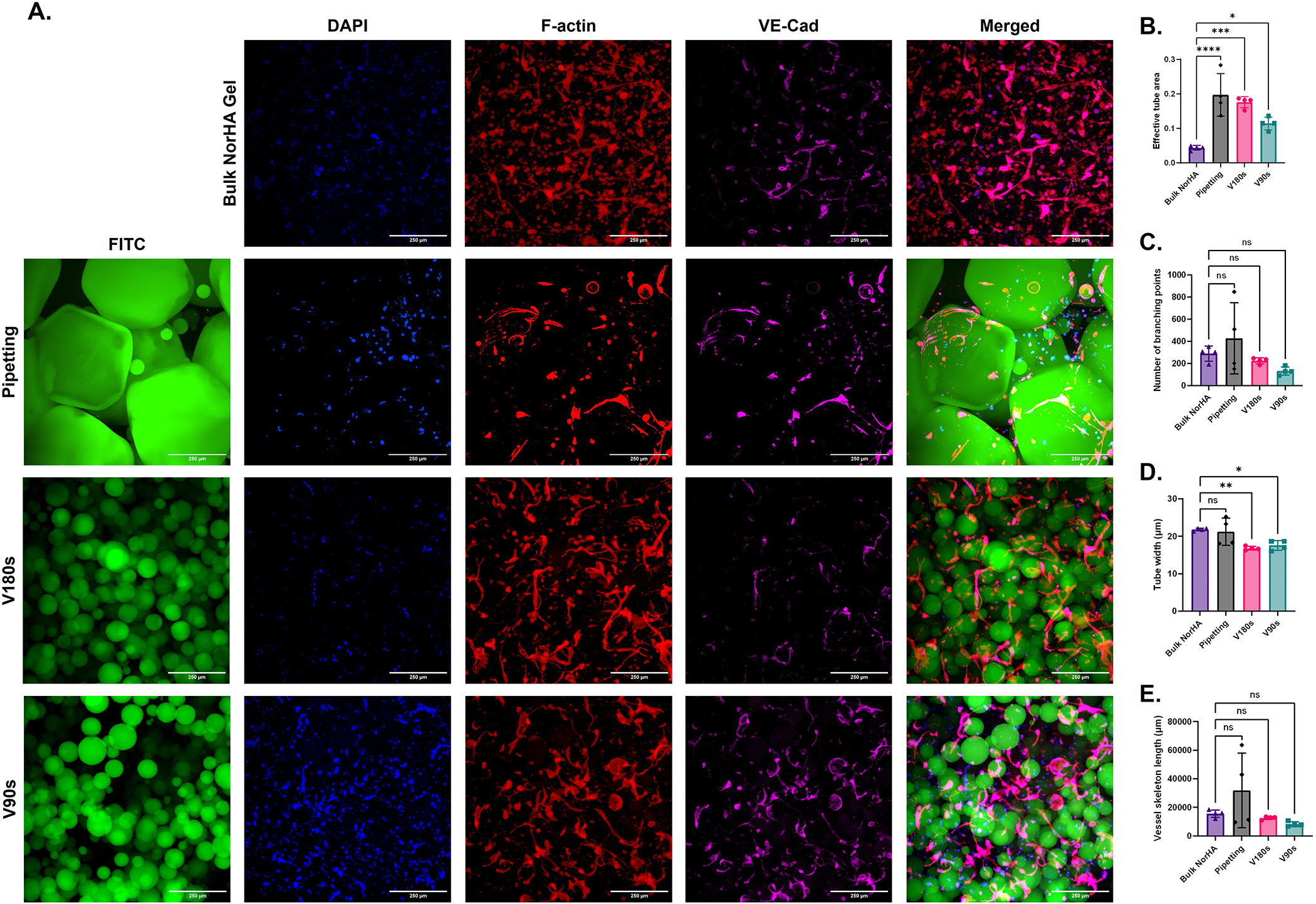
A) Z-projection for the LECs embedded in bulk NorHA and granular hydrogels produced via pipetting, vortexing 90 s, and vortexing 180 s at tight packing conditions. The bulk NorHA and the interstitial matrix of the granular hydrogels were made with 5 mm RGD and 1.2 mm MMP-sensitive crosslinker. Projections were generated using the standard deviation built-in function of ImageJ. The images show staining for DAPI (blue), F-actin (red) and VE-Cad (magenta), the green channel corresponds to the FITC-labeled microgels. The scale bars correspond to 250 μm. B) Effective degradable ECM area occupied by lymphatic capillaries. C) Number of branching points. D) Tube width. E) Vessel skeleton length. The error bars represent the mean and standard deviation. *p < 0.05, **p < 0.01, ***p < 0.001, ****p < 0.0001.

LECs were embedded within pipetting, V90s and V180s granular hydrogels at density of 8×10^6^ cells mL^−1^ based on our previous reports.^[58]^ The gels were fixed and stained for DAPI, F-actin and Vascular endothelial cadherin (VE-Cad) after 5 d under culture to be further analyzed via confocal imaging. Z-stacks for the stained gels were captured every 10 μm and the images were rendered using ImageJ. From the confocal images, no tube formation was observed for the loose packing samples (Figure S8, Supporting Information) as there was no formation of continuous cellular structures as indicated by the VE-Cad staining showing a lack of cell-cell adhesion. Instead, it was observed that the cells tend to attach to the microgels adapting to their shapes. Conversely, for the tight packing conditions (Figure 4A), we observed the formation of lymphatic capillaries with cell-cell attachment. Visually, the percentage of cells included in these networks is higher for the granular hydrogels than for the bulk NorHA, with the V180s sample having the highest vessels density.

To verify this, the capillary-like structures were quantified using the AutoTube Software.^[65]^ First, the area occupied by the lymphatic networks was analyzed (Figure 4B) and normalized based on the degradable portion of the gel (effective tube area). The results showed that the V180s and pipetting gels had the largest effective area occupied by the lymphatic networks but with a higher experimental consistency for the V180s as observed from the error bars. Importantly, the tube diameter is about 20 μm (Figure 4D) for all granular gels, which is comparable to the mean pore size of V180s sample and much smaller than the mean pore size of the pipetted gel (Figure 2D). For this reason, the gel-supported capillaries wrap around the droplet and assume the curvature of the droplets, which is clearer in the vortexing samples. Other features such as the number of branching points (Figure 4C) and the skeleton length (Figure 4E) exhibited no significant differences between the granular hydrogels and the bulk NorHA control. However, the mean skeleton size appeared substantially higher for the pipetting condition. This last point may be related to other morphological differences that can result in higher capillaries connectivity, and therefore higher complexity of vessels clusters. To prove this, additional image analyses were carried out to account for the capillaries clusters connectivity.

### 2.5. Evaluation of Lymphatic Capillaries Curvature and Clusters Connectivity

**Figure 5.**
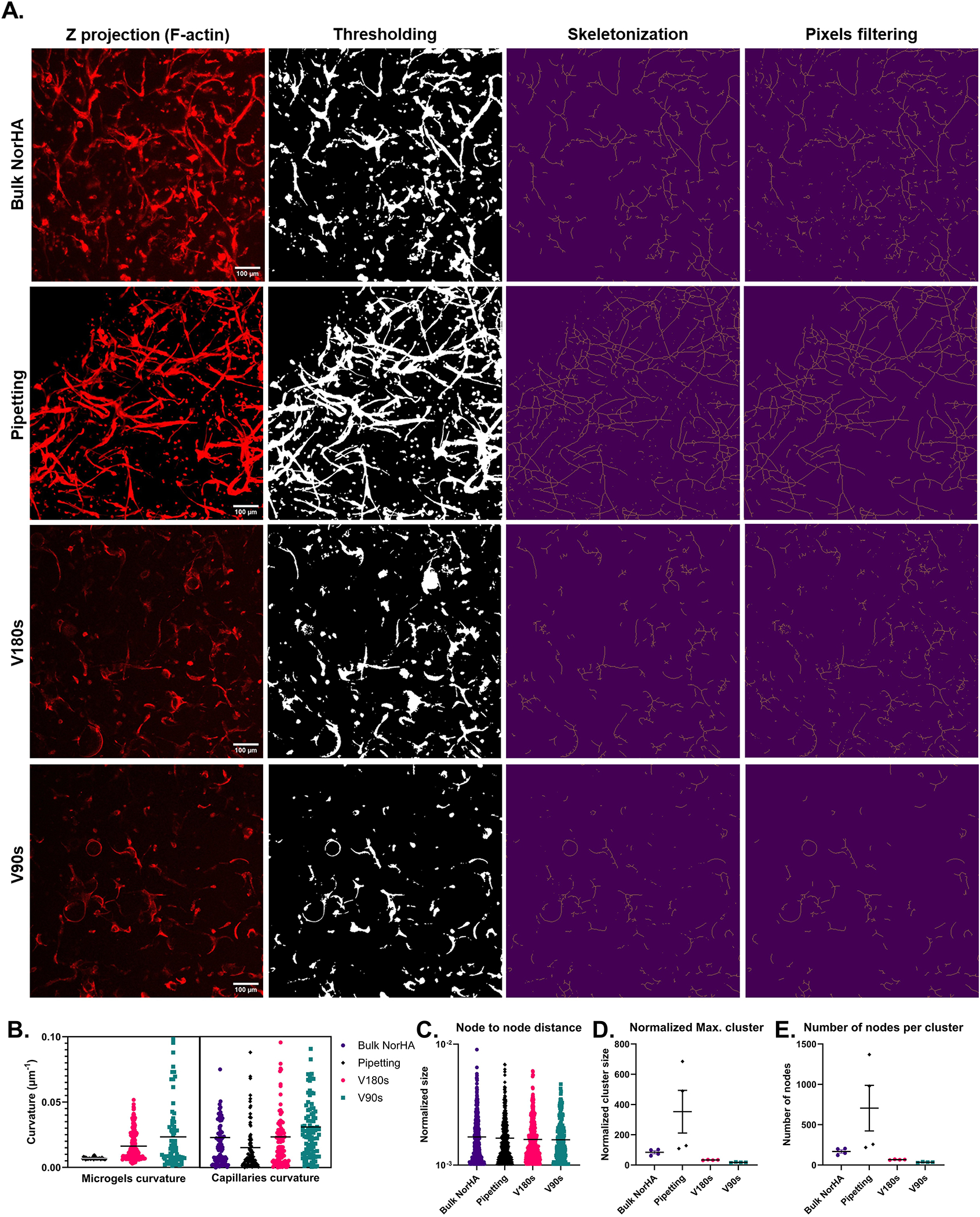
A) The schematization of the process followed for image analysis. The raw z-max projection for the F-actin channel was binarized and thresholded, then a skeletonization was carried out and finally the images were filtered using a fixed 10 μm segment threshold to remove the noise. B) Lymphatic capillaries curvature distribution. The curvature is taken to be the inverse of the average radius of curvature between two nodes. C) Normalized capillaries segments sizes. D) Biggest interconnected lymphatic cluster. E) Average number of nodes per cluster. The scale bar is 100 μm. The error bars represent the SEM.

While the effective area occupied by lymphatic vessels is larger for the granular gel samples (Figure 4), conventional methods to quantify vessels structures do not account for the connectivity of capillary structures. Hence, we carried out additional image analysis to address the connectivity of the vessels constructs. The F-actin channel was utilized to create max intensity projections of the gel samples z-stacks (Figure 5A). Afterwards, the images were binarized and thresholded for carrying out a skeletonization rendering of the lymphatic capillaries with a fixed 1-pixel width. Finally, the skeletonized images were filtered to remove structures smaller than 10 μm and decrease the noise. After quantifying capillary curvature, node-to-node segment sizes, and cluster sizes, we observed that the log-transformed data exhibited non-Gaussian distributions with long tails (Table S3, Supporting Information). Examination of these distributions revealed two distinct self-similar structures: one at the microscale (≈10 μm) between nodes and another at the macroscale (>100 μm) for cluster curvature.

Interestingly, while the node-to-node distance of capillaries across all gels followed a self-similar power-law decay, averaging 17 μm (Figure 5C), their curvature distributions were markedly different (Figure 5B). The vortexed gels exhibited multi-peak curvature distributions, reflecting their heterogeneous microgel size (Figure 2B). In contrast, the pipetted gels, despite their narrower microgel size distribution, followed a power-law capillary curvature distribution akin to the universal node-to-node distribution.

This correlation aligns with the F-actin persistence length of 17 μm,^[66]^ which provides a curvature cutoff of 0.05 μm^−1^. Below this threshold, the capillary and gel curvature distributions exhibit strong similarity, sharing local maxima at 0.01, 0.025, and 0.04 μm^−1^. Beyond 0.05 μm^−1^, capillary curvature values scatter, particularly for the V90 gel, which exhibits a long-tailed high-curvature distribution. This suggests that capillaries wrap around smaller gel droplets, aligning with visual observations that they form small, unconnected clusters instead of bridging larger voids.

This phenomenon is further confirmed by cluster size and node number measurements across four gel types, including bulk gels (Figure 5D,E). The largest clusters in pipetted gels exhibit at least a four-fold increase in size and up to a ten-fold increase in node number compared to vortexed gels. These findings suggest that gel topology, particularly size heterogeneity, plays a crucial role in promoting large capillary cluster formation with self-similar power-law distributions in both node separation and curvature.

### 2.6. Effect of Granular Hydrogels Morphology on the Key Lymphatic Markers

**Figure 6.**
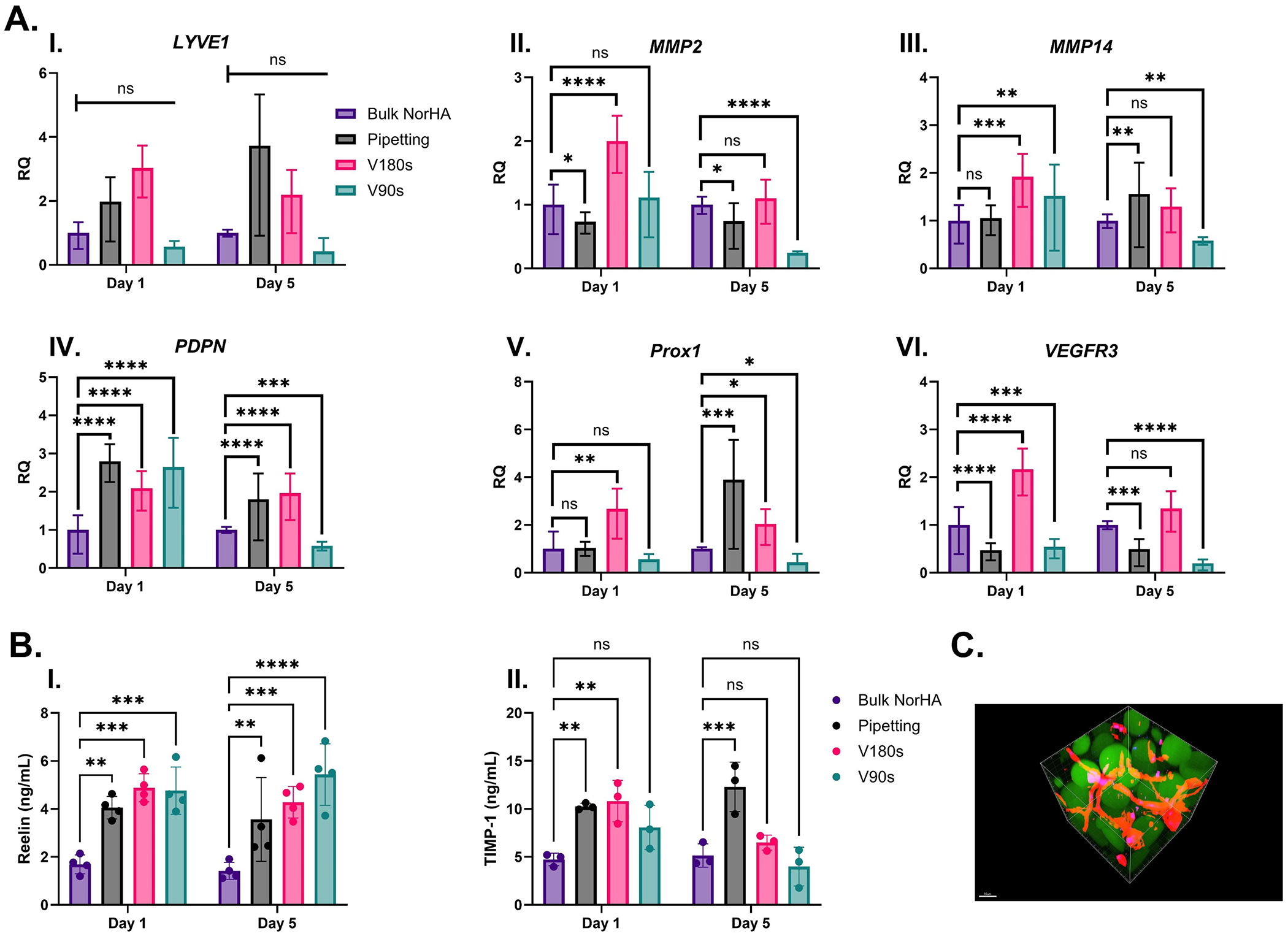
A) LEC gene expression analysis for *LYVE-1*, *MMP2, MMP14, PDPN*, *Prox1, and VEGFR3* after culture on the granular hydrogels for 24 h (Day 1) and 120 h (Day 5) with 100 ng mL^−1^ of VEGF-C and 50 ng mL^−1^ FGF. Bulk NorHA was used as reference for applying the ^ΔΔ^Ct method, while *GAPDH* was used as the housekeeping gene. B) Reelin and TIMP-1 proteins quantification at day 1 and day 5. C) 3D rendering of V180s gel (green—FITC) exhibiting lymphatic capillaries networks formation (red—F-actin). The error bars represent the mean and standard deviation. *p < 0.05, **p < 0.01, ***p < 0.001, ****p < 0.0001.

The gene expression levels for key lymphatic markers were analyzed using RT-qPCR and quantified using the ^ΔΔ^Ct method with *GAPDH* as housekeeping gene, and monolayer LECs and bulk NorHA as reference. Gels were collected at day 1 and 5 to be mechanically homogenized for RNA isolation. After cDNA synthesis, the samples were assessed using RT-qPCR. Although some of the lymphatic markers were upregulated in the monolayer-cultured LECs compared to all the gel samples (Figure S9, Supporting Information), as the LECs cultured in tissue culture plastic surface are unable to form lymphatic capillaries, bulk NorHA was chosen as the control sample (Figure 6A).

*LYVE1* exhibited no significant changes across all the gel samples. Nonetheless, *PDPN* was upregulated in all the granular gel samples on day 1, and at day 5 except for the V90s hydrogel. *LYVE-1* and *PDPN* are important lymphatic markers, responsible for leukocyte trafficking and LEC recognition, respectively.^[67–69]^ Remarkably, the key receptor to vascular endothelial growth factor-C (VEGF-C), *VEGFR3*, is upregulated just for V180s sample on day 1, but reaches a similar level as the bulk NorHA control on day 5. Similarly, prospero homeobox 1 (*Prox1*) shows a ≈2-fold upregulation for the V180s gel on day 1 and on day 5, whereas the pipetting gel experiences upregulation on day 5. Since *Prox1* is the master regulator of lymphatic vasculature, these results indicate lymphatic vessel phenotype.^[70,71]^

These results, combined with a similar late upregulation of *MMP14* in the pipetting gel compared to the V180s gel, account for the faster maturation time in the V180s gel compared to the other gel samples. This is also supported by the 3-fold and ≈2-fold upregulation of *MMP2* and *MMP14* early on, which plays a major role in angiogenesis.^[57,72,73]^ It is worth mentioning that *Prox1* expression levels follow a similar trend as the *MMP14*. This suggests that the upregulation of *MMP14* derived from the ECM remodeling and vessels formation could trigger *Prox1* upregulation to maintain lymphatic homeostasis.^[56,74]^

To further confirm how the differences in vessel maturation may affect the LECs function, Reelin and Tissue Inhibitors of Metalloproteinase (TIMP-1) secretions were quantified through ELISA (Figure 6B). Reelin is a key lymphangiocrine, which plays a major role in lymphangiogenesis and promote the health of many vital organs.^[75,76]^ Reelin is an indicator of tight junctions of LECs with functional architecture, as its secretion depends on the presence of VE-cadherin.^[77,78]^ Quantification of reelin secretion showed higher levels for the granular hydrogels, suggesting that the encapsulated LECs have the desired functionality with the formation of junctions which are necessary for the generation of functional and mature lymphatic capillaries. Moreover, TIMP-1 functions to inhibit the activity of MMPs, which indicate vessel maturation and stabilization.^[23,64,79]^ TIMP-1 quantification showed an increase in TIMP-1 secretion over time, with the V180s gel having a slightly higher earlier production compared to the pipetting gel. Similarly to the trends observed in the gene expression levels, this trend shifts at day 5, suggesting once more an earlier vessel formation in the V180s gel.

## 3. Conclusion and Future Directions

This study presents novel insights into factors influencing lymphangiogenesis within granular hydrogels, enabling the generation of early-stage lymphatic sprout formation without the need for any supporting cells (i.e., fibroblasts). We assessed granular hydrogels morphology and their effects on lymphatic capillaries and key lymphatic markers. The various microgel generation methods (pipetting and vortexing) using NorHA polymer resulted in distinct morphologies. Vortexing-produced gels exhibited higher porosity but wider microgel size distribution, leading to tighter packing compared to pipetting, resulting in smaller pores. This morphology resulted in a higher storage modulus and wider LVR ranges due to increased interparticle contact in loose packing conditions. Such differences were drastically reduced when the granular gels were produced at tight packing conditions.

Hydrogels’ morphological variances significantly affected the lymphatic development. While no lymphatic sprouting was observed for the loose packing samples, at tight packing conditions the pipetting and vortexing gels exhibited different vessel formation and maturation patterns, with the V180s gel having earlier development and maturation. Nonetheless, we found that the lymphatic capillaries have a higher connectivity in the pipetting gels with a lower number of satellite small vessel constructs. Remarkably, this enhanced connectivity might be derived from the lower mean curvature of the pipetting templates compared to the vortexing ones which reduce the cell-microgels contact. This lower curvature of the pipetting template is a result of the highly regimented topology of the gel droplets, with highly monodispersed size distribution. Quantitative RT-PCR data supported these observations, indicating an early *MMP2* and *MMP14* up-regulation in the V180s gel with a higher presence of TIMP-1 protein, which indicates lymphatic vessel maturation and stabilization. Moreover, the secretion of reelin, key lymphangiocrine, indicates lymphatic vessel functionality for all the granular gel samples, that can be further used to support co-culture of other vital organs for tissue engineering applications.^[75]^

Overall, this study provides new insights into 3D in vitro lymphatic tube formation, which contributes to our understanding of lymphatic biology and may lead to novel approaches to lymphatic regeneration. Importantly, lymphatic tube networking seems to exhibit some innate curvature and length scales such that robust capillary network is promoted by a templating granular gel that exhibits the same length scales and curvatures. Such important design parameters can be incorporated into future approaches in lymphatic tissue regeneration.

## 4. Experimental Section

### NorHA Synthesis:

NorHA polymer was synthesized following established procedures.^[33,58,80]^ Briefly, hyaluronic acid (HA) in its tetrabutylammonium (HA-TBA) form was dissolved in anhydrous dimethyl sulfoxide (DMSO). Dimethyl aminopyridine (DMAP), norbornene-2-carboxylic acid, and di-tert-butyl dicarbonate (Boc2O) were added to the solution and were allowed to react for 20 h. The NorHA product was dialyzed for two days with a NaCl solution in DI-water, and two days more with DI water alone. The polymer solution was then frozen at −80 °C, lyophilized for one day, and stored at −20 °C prior to usage. To quantify the degree of substitution (DS), lyophilized polymer was dissolved in deuterium oxide (D_2_O) and analyzed via ^1^H -NMR (Bruker AVANCE III HD 400 Nanobay).^[80]^

### Granular Hydrogels Fabrication:

For the fabrication of the granular gels, first NorHA was dissolved (2% w/v) in 1X Dulbecco’s phosphate-buffered saline (DPBS) (Corning Life Sciences, MA, USA) with DL-dithiothreitol (DTT) (Sigma-Aldrich, LO, USA) and FITC (GenScript, NJ, USA) at a ratio with respect to the norbornene groups of 0.8 and 0.01, respectively. The water soluble photoinitiator 2-hydroxy-4′-(2-hydroxyethoxy)-2-methylpropiophenone (Irgacure 2959) was used at 0.05% (w/v).

The microgels were fabricated using two different alternatives 1) vortexing, and 2) pipetting. In all cases, microgels were produced via emulsification using a 008-FluoroSurfactant (1 weight%) dissolved in HFE7500 (RAN Biotechnologies, Inc, MA, USA). For vortexing the prepared polymer solutions were mixed in a 1:1 ratio with the oil and stirred at 60 s, 90 s, and 180 s at 2500 rpm.

For pipetting a home-made pipette microfluidic device was used for the microdroplets generation. Elliptical pipette tips were fabricated following established procedures. Tips were deformed with a torque screwdriver using 20 inch-pounds and equipped with a multi-channel pipettor. A stepper motor-controlled system was used to dispense the polymer solution inside the oil (Figure S2 of the Supporting Information). Different aspiration/dispensing cycles and flow rates were used for generating microgels.

Upon collection, the microdroplets were exposed to UV light (Omnicure S2000, Excelitas Canada Inc., Canada) with a power of 20 mW cm^2^ for 4 min to prompt their gelation via thiol-ene reaction.^[6]^ Moreover, 30% 1H,1H,2H,2H-perfluoro-1-decanol (Sigma-Aldrich, LO, USA) in HFE7500 was mixed in a 1:1 ratio with the suspended microgels for breaking the emulsion. The suspension was centrifuged at 600 *g* for 5 min and the oil phase was retrieved afterwards. The microgels were then washed and centrifuged at 1500 *g* two times with ethanol (70% purity) and seven times with DPBS to extract the remaining oil. The microgels were filtered using a 37 μm reversible cell strainer (STEMCELL Technologies, Canada) to separate the excess DPBS. Finally, loose and tightly packed granular gels were fabricated by centrifuging the microgels at 1500 *g* and 6000 *g*, respectively. All the materials were sterilized prior to their implementation in cell culture.

### Morphological Characterization of the Gels:

Fluorescence microscopy (ECHO Revolve, San Diego, CA-USA) was utilized to take micrographs of a monolayer of microgels suspended in 008-FluoroSurfactant following previously reported procedures.^[62]^ Confocal microscopy was performed using a Nikon AX-R (Nikon Instruments Inc, NY, USA) to obtain randomized 3D stacks of the granular gels. A 10x objective was used for a Z-stack range of roughly 400 μm with an interslice spacing of ≈7 μm. The stacks were smoothened and thresholded, while the porosity and pores cross section were estimated by counting the binary pixels using a Python code. ImageJ was used to determine the size distribution of the microgels with the analyze particles built-in function. Similarly, the pore size and diameter were estimated as described in previous papers.^[27]^ The 3D stacks were then rendered using the Nixon-NIS Elements Software to obtain the orthogonal view of the granular gel.

### Rheological Characterization:

Dynamic rheological measurements were carried out using a Discovery HR-2 Rheometer (TA Instruments, DE, USA) equipped with a solvent trap. A 20 mm plate-plate geometry for all the experiments. Silicon oil was placed along the edge of the plate after sample trimming. Amplitude sweep tests were performed at 10 rad s^−1^ with a strain ranging from 0.1% to 130%. After determining the linear viscoelastic region (LVR) frequency sweep tests from 0.1 to 20 rad s^−1^ at a constant 1% strain were performed. Additionally, stress relaxation experiments were carried out for 3 min under 1% strain. Finally, cyclic step strain analyzes were performed by repeatedly cycling the strain for 60 and 30 s from 1% to 800% for the loose packing gels, and 1% to 400% for the tight packing gels, to evaluate the self-healing behavior of the granular gels. A 600 μm GAP was used for all the rheological measurements and no wall-slip was evidenced while carrying out the tests. All the rheological measurements were carried out in triplicate at 25 °C and the collected data was smoothed using the Savitzky-Golay filter before plotting.

### Cell Culture:

Human juvenile LECs derived from the foreskin of four donors (C-12216, PromoCell, Heidelberg, Germany) were expanded and used for experiments between passages 4 and 8, as previously described.^[56–58]^ Briefly, LECs were grown in endothelial cell growth media MV2 (EGM-MV2, C-22022, PromoCell, Heidelberg, Germany) and incubated at 37 °C with 5% CO_2_. To keep the cell passaging constant throughout experiments, cells were passaged every 5 d at a 1 to 3 ratio. Human LECs were characterized by the positive expression of *CD31*, *LYVE-1*, *Prox1*, and *PDPN* throughout the experiments. Cell lines were routinely tested for mycoplasma contamination and were negative throughout the present study.

### Lymphatic Sprouting Assays:

A degradable NorHA polymer solution was used to generate as an interstitial matrix that was then mixed with each one of the non-degradable granular gels. The polymer solution used as the interstitial matrix precursor was prepared using 2% w/v of NorHA alongside 5 mm thiolated RGD (GCGYGRGDSPG, molecular weight [MW]: 1025.1 Da, GenScript,^[39]^ 1.2 mm thiolated MMP-sensitive crosslinker (GCRDGPQG↓IWGQDRCG, MW: 1754.0 Da; down arrow indicates the site of proteolytic cleavage, GenScript), and 0.05% w/v Irgacure 2959. Cells were added to the polymer solution and suspended by pipetting at a density of 8 × 10^6^ cell mL^−1^. The cell-laden polymer solution was mixed with the granular gels through pipetting with wide bore tips. The volumetric ratio of the NorHA solution added to the granular gels was adjusted to match their calculated porosity. The mixtures were placed in a glass bottom 96-well plate where these were exposed to UV light with a power of 10 mW cm^2^ for 1 min to prompt the interstitial matrix gelation. Bulk NorHA gels fabricated utilizing the same composition used for the degradable interstitial matrix were used as experimental control. The constructs were cultured using MV2 media with 100 ng mL^−1^ of VEGF-C (R&D Systems, MN, USA) and 50 ng mL^−1^ FGF (R&D Systems). Constructs were cultured for 5 d with media changes every day and micrographs were taken daily using bright field microscopy to monitor vessels development. The porosity of the granular gels was recalculated to assess the effect of the filling matrix addition.

### Immunostaining and Imaging:

Constructs were washed with DPBS and fixed with 3.7% PFA. Furthermore, the samples were blocked with 1% BSA, permeabilized with 0.1% Triton-X and stained for F-actin (Phalloidin-iFluor 594, Abcam, MA, USA) DAPI, and VE-Cadherin (conjugated with Alexa Fluor 647, Santa Cruz Biotechnology, TX, USA) to visualize lymphatic tube formation (Table S1, Supporting Information). Confocal microscopy was performed to obtain 3D stacks of the lymphatic vessels. 10x and 20x objectives were used for a Z-stack range of roughly 400 μm with an interslice spacing of ≈10 μm.

### Lymphatic Networks Analysis and Quantification:

The AutoTube Software was used to analyze and quantify the lymphatic networks in terms of occupied area, skeleton size, tube width, and number of branches.^[58]^ For this, the z-stacks obtained with the 10x objective were preprocessed by first obtaining the Z max intensity projection, and then by smoothing and denoising. Finally, thresholding was applied to exclude cells that were not forming vessels from the quantification. The calculated network area was normalized according to the degradable portion of each of the gels. Finally, the z projections of the 20x Z-stacks were rendered using the ImageJ standard deviation built-in function after smoothing, denoising, and color thresholding.

### Lymphatic Related Genes Expression:

Granular hydrogels were collected at day 1 and 5 to be mechanically homogenized for RNA extraction and purification. RNA was reverse transcribed using a High-Capacity cDNA reverse transcription toolkit (Thermo Fisher, MA, USA) as previously described.^[81]^ TaqMan Universal PCR Master Mix was used with the cDNA to determine the gene expression levels for the genes of interest (Table S2, Supporting Information). *GAPDH* was used as endogenous control for the relative expression which was analyzed through the ^ΔΔ^Ct method. Both monolayer cultured LECs and Bulk NorHA were used as controls and reference in the ^ΔΔ^Ct estimations. All the samples were prepared in triplicate.

### Reelin and TIMP-1 Proteins Quantification:

To carry the Reelin and TIMP-1 protein quantification, gel culture supernatants were collected at day 1 and 5 and centrifuged and filter through 0.2 μm filters. Then, bradford assays (Thermo Fisher, MA, USA) were performed to quantify the protein content in each sample. Abcam Elisa kits were used to quantify Reelin (ab284620—Abcam, MA, USA) and TIMP-1 (ab100651). The experiments were carried out following the manufacturer’s suggested procedure. Each sample was run at least in triplicates.

### Statistical Analysis:

Data visualization and analysis were carried out using GraphPad Prism software. The reported experiments were repeated at least three times. Shapiro-Wilk test was used to test the normality of sample sizes <50 n, while Kolmogorov-Smirnov test was used for *n* ≥50. Welch’s t test was utilized to compare differences between two sample groups, while one-way Brown-Forsythe and Welch ANOVA tests were carried out to compare differences between more than two groups. The corresponding levels of significance were *p < 0.05, **p < 0.01, ***p < 0.001, ****p < 0.0001.

## Supplementary Material

Supplementary Material

Supporting Information is available from the Wiley Online Library or from the author.

## Data Availability

The data that support the findings of this study are available in the supplementary material of this article.
