# Supplementary material for: Tuning the Morphological Properties of Granular Hydrogels to Control Lymphatic Capillary Formation
Source: Adv Mater Interfaces. Author manuscript; Available in PMC 2026 Jul 29. (PMC13410779; doi:10.1002/admi.202401037)
Supplement: Supplementary Material [file NIHMS2162922-supplement-Supplementary_Material.docx]

**Supplementary Information for:**

**Tuning the Morphological Properties of Granular Hydrogels to Control Lymphatic Capillary Formation**

Daniel Montes^1,2^, Sanjoy Saha^1,3^, Angela Taglione^2^, Donghyun Paul Jeong^1,3^, Liao Chen^1,2^, Fei Fan^1,3^, Hsueh-Chia Chang^1,2,3^, Donny Hanjaya-Putra^1,2,3,4^

^1^ Bioengineering Graduate Program, University of Notre Dame, Notre Dame, IN 46556

^2^ Chemical and Biomolecular Engineering, University of Notre Dame, Notre Dame, IN 46556

^3^ Aerospace and Mechanical Engineering, University of Notre Dame, Notre Dame, IN 46556

^4^ Center for Stem Cell and Regenerative Medicine, University of Notre Dame, Notre Dame, IN 46556

**This Supplementary Information includes:**

Supplementary Table 1-3

Supplementary Figure S1 to S10

**Supplementary Table 1. Antibodies used for this study.**

| **Name of Reagent** | **Company** | **Host Species** | **Catalog Number** | **Dilution Factor** |
| --- | --- | --- | --- | --- |
| Phalloidin | Thermo Fisher | iFluor 594 | ab176757 | 1:1,000 |
| VECAD | Santa Cruz | Alexa Fluor 647 | sc-9989 AF647 | 1:50 |

**Supplementary Table 2. Primers used for this study.**

| **Genes** | **Catalog Number** |
| --- | --- |
| *LYVE-1* | Thermo Hs00272659_m1 |
| *PDPN* | Thermo Hs00366766_m1 |
| *Prox-1* | Thermo Hs00896294_m1 |
| *MMP2* | Thermo Hs01548727_m1 |
| *MMP14* | Thermo Hs01037003_g1 |
| *VEGFR3* | Thermo Hs01047677_m1 |
| *GAPDH* | Thermo Hs02786624_g1 |

**Supplementary Table 3. Kolmogorov-Smirnov Analysis.** Kolmogorov-Smirnov (KS) Analysis normality analyzes carried out for the curvature, segment size and cluster size of the lymphatic capillaries cultured in Bulk NorHA and the Pipetting, V180s and V90s granular hydrogels. The KS distance shows that the datasets are non-Gaussian distributions.

| **KS distance raw data** | | | | |
| --- | --- | --- | --- | --- |
| **Analysis** | **Bulk NorHA** | **Pipetting** | **V180s** | **V90s** |
| Curvature | 0.2112 | 0.2348 | 0.2122 | 0.1322 |
| Segment size | 0.1307 | 0.1301 | 0.1359 | 0.1094 |
| **KS distance Log transformed data** | | | | |
| **Analysis** | **Bulk NorHA** | **Pipetting** | **V180s** | **V90s** |
| Curvature | 0.0898 | 0.04321 | 0.115 | 0.1343 |
| Segment size | 0.1307 | 0.1301 | 0.1359 | 0.1094 |


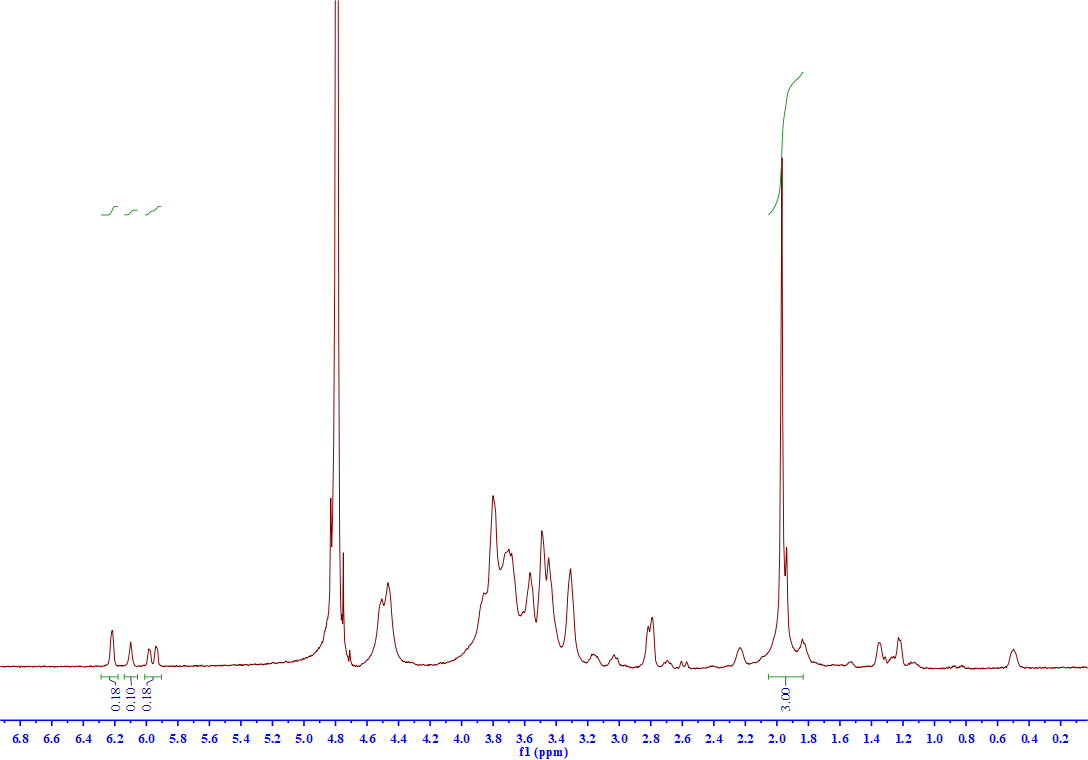


**Figure S1.** ^1^H NMR spectrum of NorHA with DS value 22%.


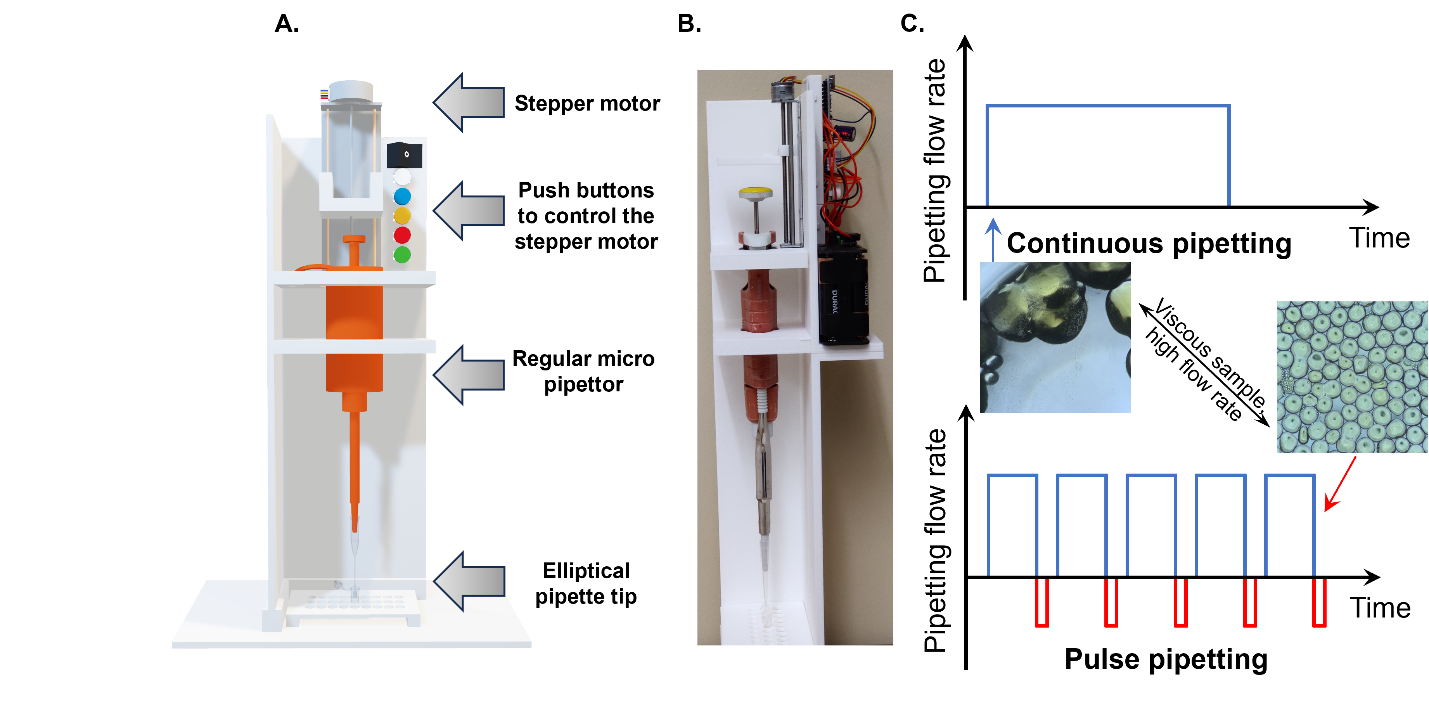


**Figure S2.** Scheme of the automated system used for producing the microgels via pipetting. **(A)** Sketch of the system adapting a stepper motor for controlling the aspiration/dispensing of the samples by pulsing a regular micro-pipettor. The system includes four push bottoms programed via Arduino to control the stepper motor. **(B)** Real photograph of the pipetting system set up. **(C)** Representation of the effect of the aspiration/dispensing cycles on the morphology of the produced droplets.


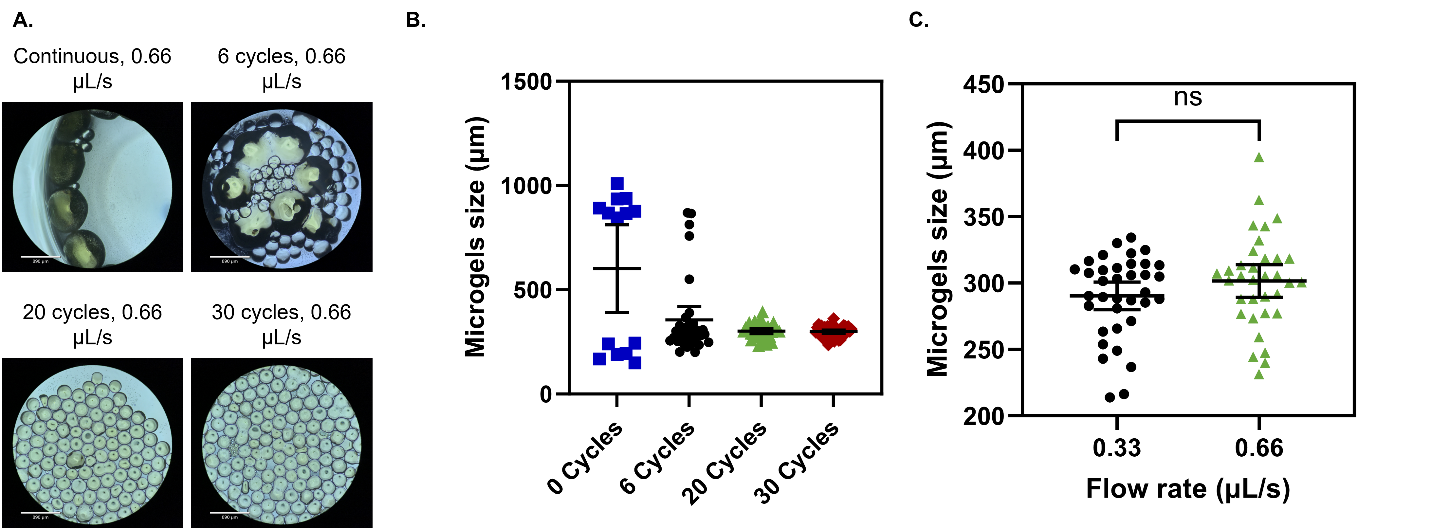


**Figure S3.** Size distribution analysis for the microgels generated by Pipetting. **(A)** micrographs for the microgels produced by pipetting with different aspiration/dispensing cycles (0, 6, 20 and 30 cycles). The scale bar is 890 µm. **(B)** Scatter plot showing microgels sizes vs the number of aspiration/dispensing cycles. **(C)** Scatter plot showing microgels sizes vs flow rate. The mean and CI (95%) are reported for all the results as error bars. [*p<0.05, **p<0.01, ***p<0.001, ****p<0.0001].


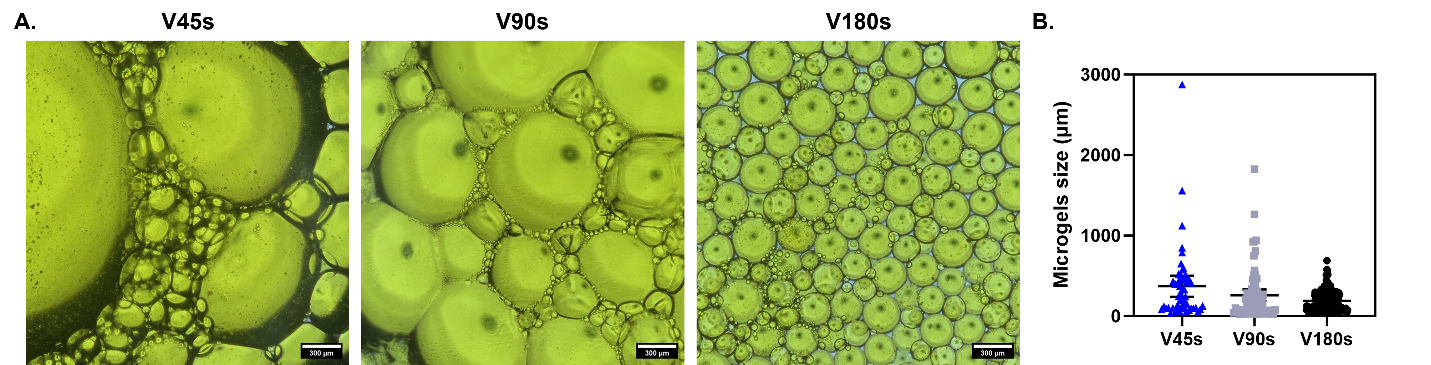


**Figure S4.** Size distribution analysis for the microgels generated by vortexing. **(A)** micrographs for the microgels produced by vortexing 45 s (V45s), 90 s (V90s), and 180 s (V180s). The scale bar is 300 µm. **(B)** Scatter plot showing microgels sizes. The error bars represent the mean and CI (95%).


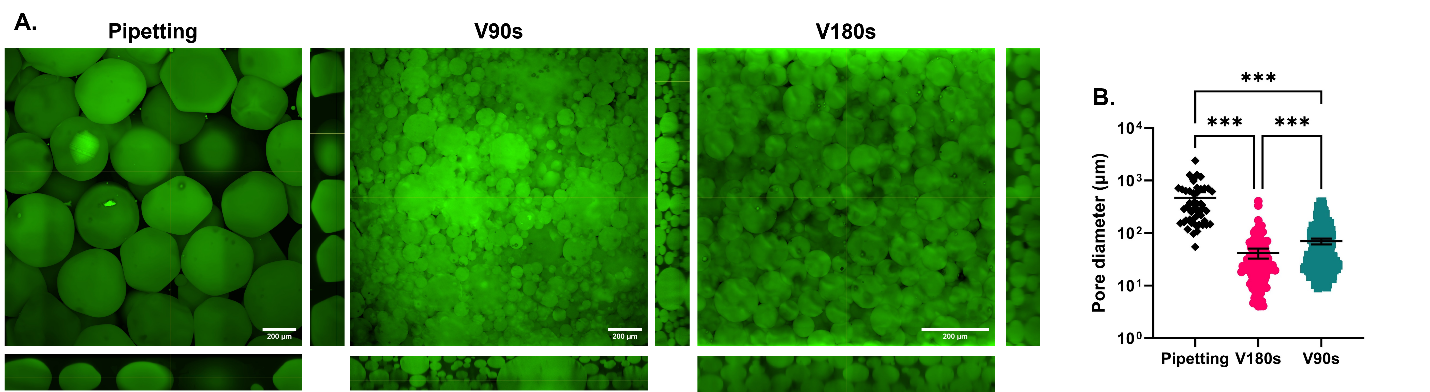


**Figure S5.** Morphological characterization of the granular gels produced by vortexing and pipetting and 1500 g. (**A**) Orthogonal view of confocal imaging of FITC labeled granular gels, and porous structure rendering, respectively for Pipetting, vortexing 90s, and vortexing 180s. (**B**) Pore cross sectional area. (**C**) Pore diameter. The error bars represent the mean and CI (95%). The scale bar is 200 µm. *p<0.05, **p<0.01, ***p<0.001, ****p<0.0001.


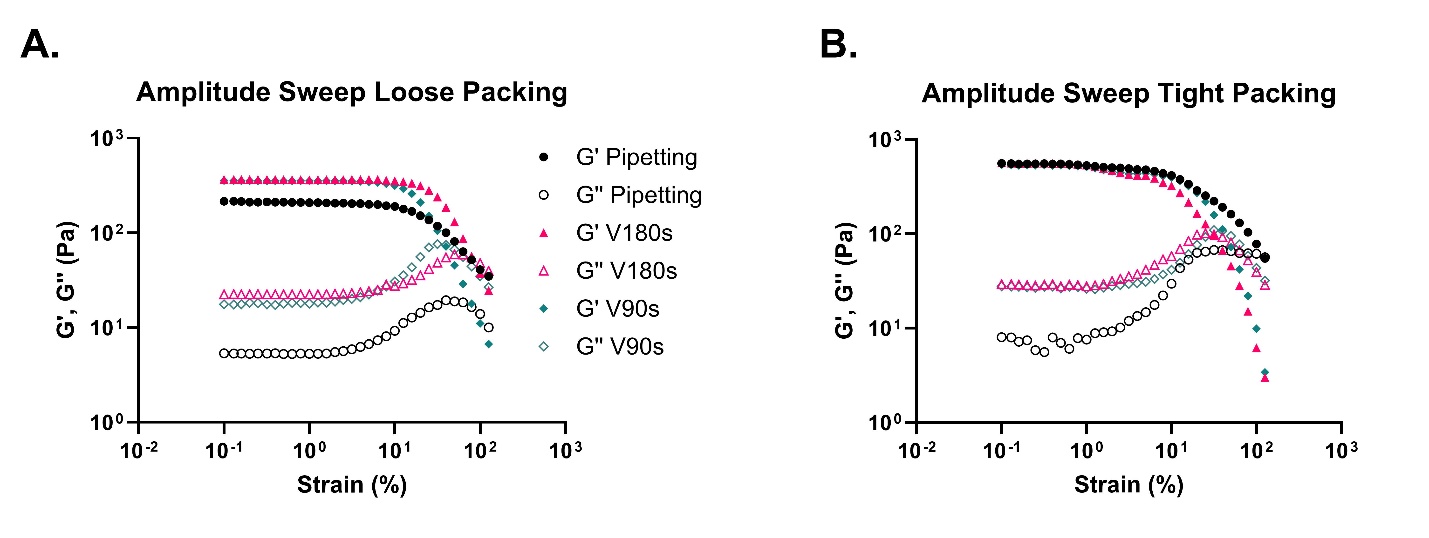


**Figure S6.** (**A**) Amplitude sweep tests measured from 1% to 130% strain at 10 rad/s for the loose packed gels. (**B**) Amplitude sweep tests measured from 1% to 130% strain at 10 rad/s for the tight packed gels. Filled symbols correspond to the storage modulus (*G’*) while the hollow symbols correspond to the loss modulus (*G’’*). All samples were measured by triplicate at 25°C.


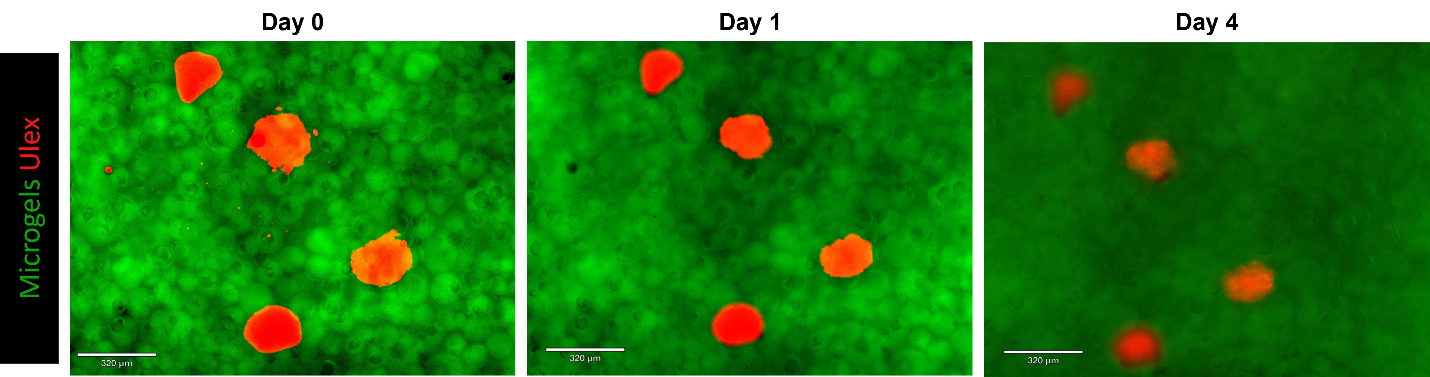


**Figure S7.** LECs spheroids cultured in vortexing-produced granular hydrogel in the absence of an interstitial matrix. Granular hydrogels were stained with FITC and lymphatic endothelial cells were stained with rhodamine-conjugated *Ulex Europaeus Agglutinin I* (UEA I).


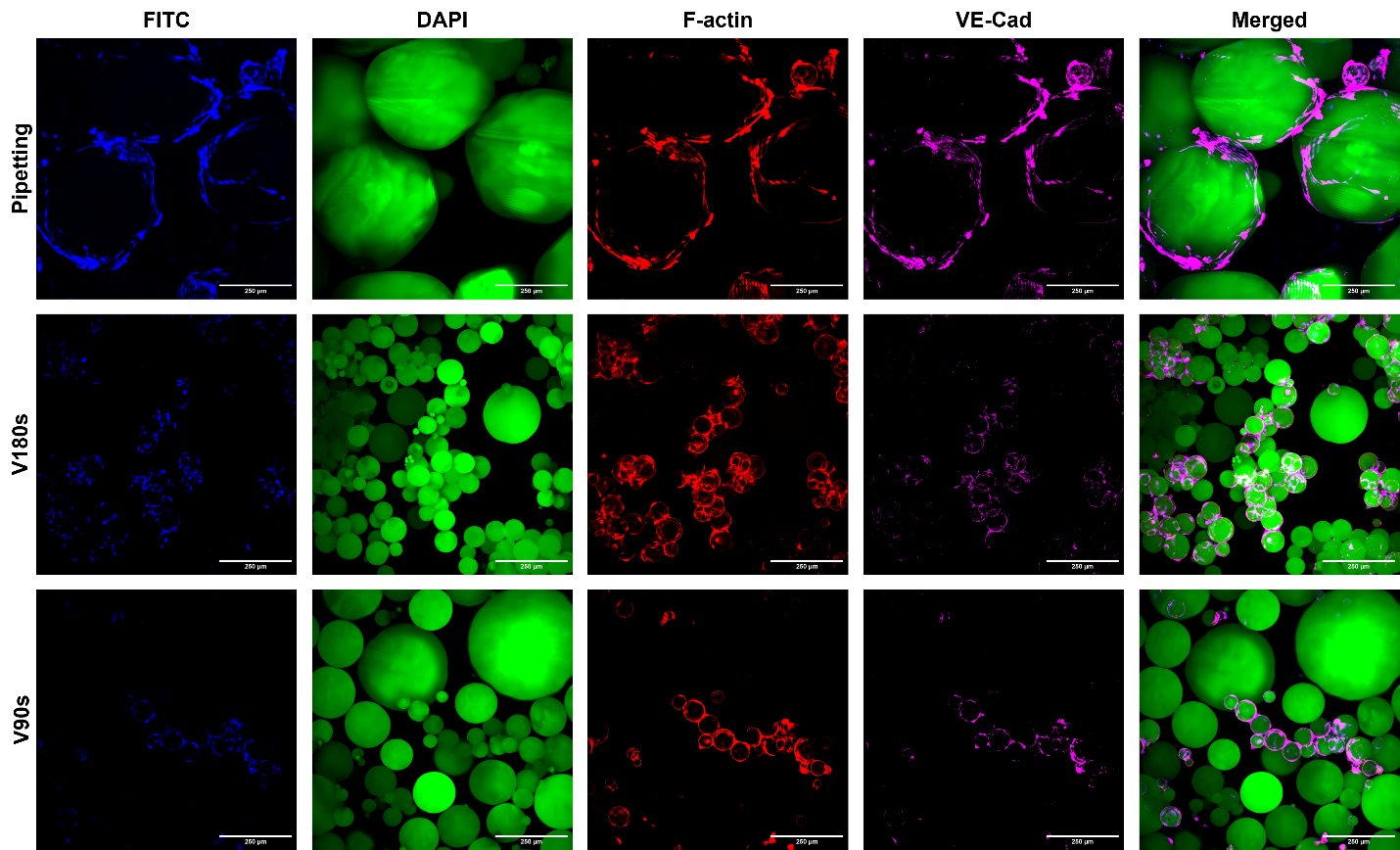


**Figure S8.** Z projection for the LECs embedded in bulk NorHA and granular hydrogels produced via pipetting, vortexing 90 s, and vortexing 180 s at loose packing conditions. The bulk NorHA and the interstitial matrix of the granular hydrogels were made with 5 mM RGD and 1.2 mM MMP-sensitive crosslinker. Projections were generated using the standard deviation built-in function of ImageJ. The images show staining for DAPI (blue), F-actin (red) and VE-Cad (magenta), the green channel corresponds to the FITC-labeled microgels. The scale bar corresponds to 250 µm.


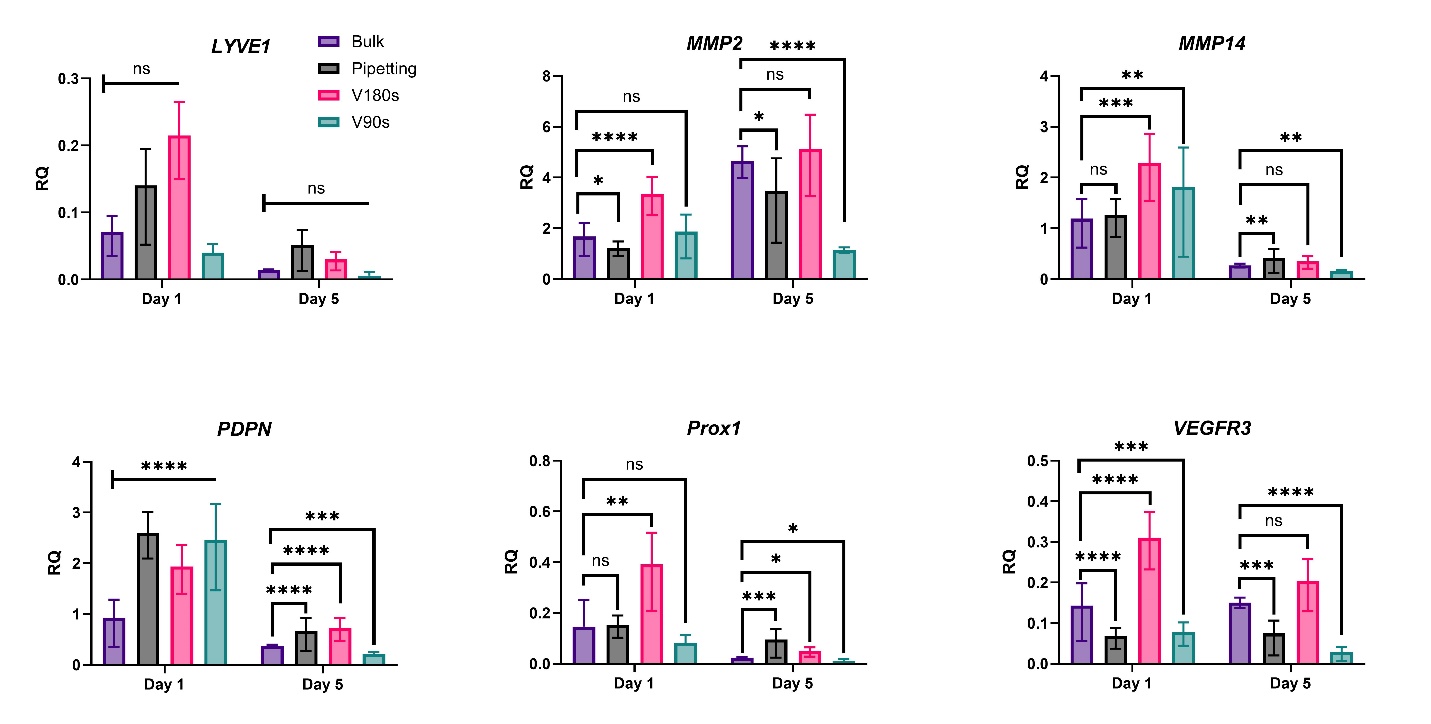


**Figure S9.** LEC gene expression analysis for *LYVE-1*, *MMP2, MMP14,* *PDPN*, *Prox1, and VEGFR3* after culture on the granular hydrogels for 24 h (Day 1) and 120 h (Day 5) with 100 ng∙mL^-1^ of VEGF-C and 50 ng∙mL^-1^ FGF. Monolayer LECs were used as reference for applying the ^ΔΔ^Ct method, while *GAPDH* was used as the housekeeping gene. The error bars represent the mean and standard deviation. *p<0.05, **p<0.01, ***p<0.001, ****p<0.0001.


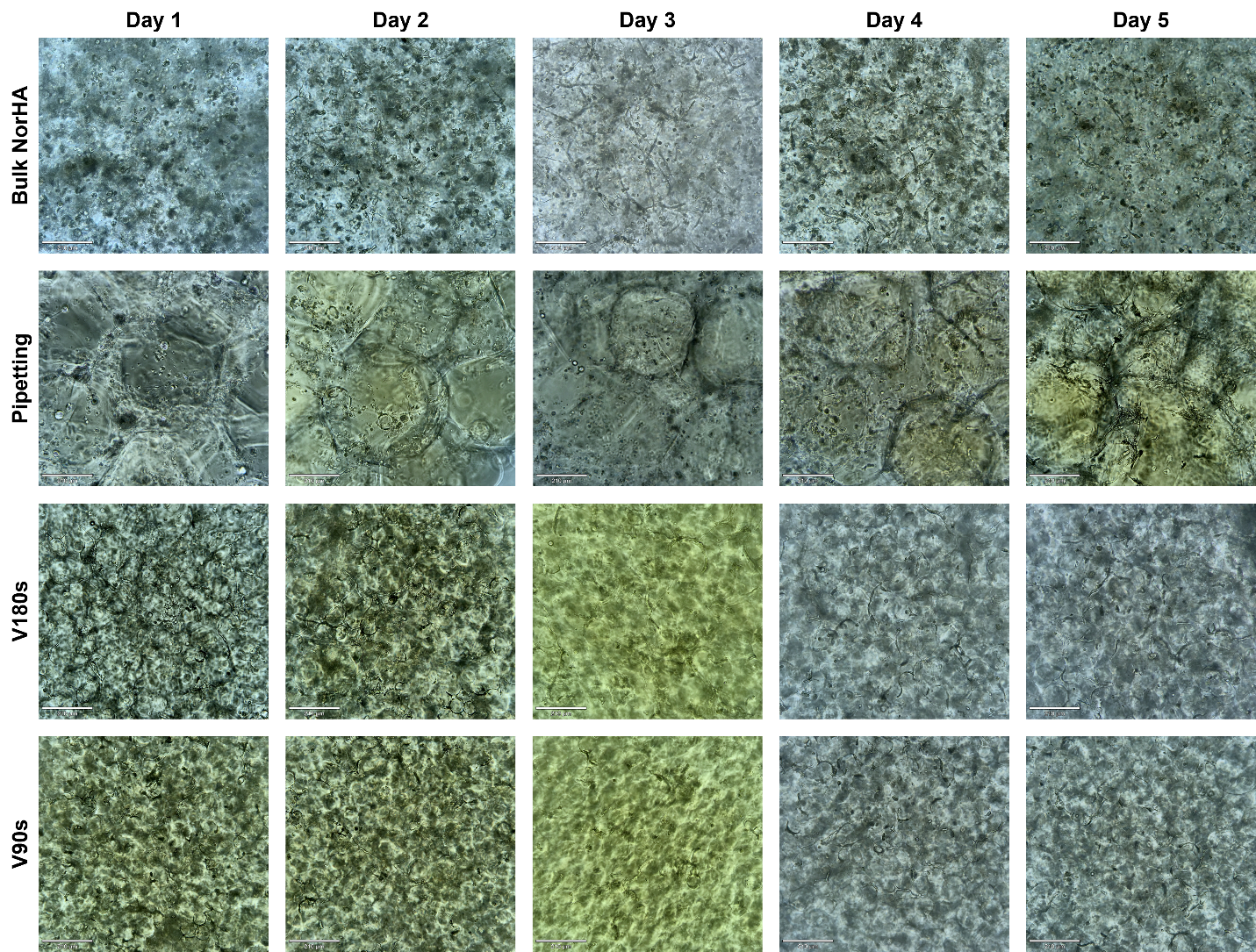


**Figure S10.** Bright field images for the pipetting granular hydrogel made with an interstitial matrix composed of 5 mM of RGD and 1.2 mM of MMP-sensitive crosslinker seeded with an LEC density of 8x10^6^ cells∙mL^-1^. Micrographs were taken from day 0 to 5 to show the morphological changes seen for the seeded cells over time. The scale bar corresponds to 210 µm.
